# Disturbance by large herbivores alters the relative importance of the ecological processes that influence the assembly pattern in heterogeneous meta-communities

**DOI:** 10.1002/ece3.987

**Published:** 2014-02-17

**Authors:** Haruka Ohashi, Yoshinobu Hoshino

**Affiliations:** 1 Faculty of Agriculture, Tokyo University of Agriculture and Technology3-5-8 Saiwai-cho, Fuchu, Tokyo, 183-8509, Japan; 2 Institute of Agriculture, Tokyo University of Agriculture and Technology3-5-8 Saiwai-cho, Fuchu, Tokyo, 183-8509, Japan

**Keywords:** Biodiversity, biotic homogenization, *Cervus nippon*Temminck, community assembly, compositional turnover, niche breadth, null model approach, overabundance, Sika deer, variation partitioning

## Abstract

Disturbance caused by large herbivores can affect the relative importance of ecological processes in determining community assembly and may cause a systematic loss of biodiversity across scales. To examine changes in the community assembly pattern caused by an overabundance of large herbivores in Japan, we analyzed community composition data from before and after the overabundance occurred. The community assembly pattern becomes more random after the deer overabundance. In addition, result of variation partitioning revealed decrease in importance of environmental processes and increase in importance of spatial processes. However, response of turnover rate, niche breadth, and niche overlap was heterogeneous, according to scale of each environmental gradient. Our results emphasize the importance of conserving habitat specialists that represent the local environment (habitat type and topography) at various altitudinal ranges to maintain biodiversity at regional scales under the increasing pressure of large herbivores.

## Introduction

Pattern of biodiversity is structured by ecological processes that operate at several hierarchical scales, from local ecosystems to landscapes and even entire biogeographic regions. Understanding how local communities assemble from the regional species pool and how biodiversity structures develop across different scales are key questions in ecology. These issues have crucial practical importance for efforts to deal with ongoing global change. Two main types of ecological processes influence the assembly of species from the regional species pool into a local community. Deterministic processes include niche differentiation and interspecific interactions, which operate to create variation among locations. Stochastic processes include colonization and extinction dynamics (MacArthur and Wilson 1967), as well as ecological drift and dispersal limitations (Hubbell 2001). In general, both types of processes occur simultaneously, and their relative importance differs among the types of meta-community (Leibold et al. 2004).

Disturbance is an ecological process that can affect the relative importance of deterministic and stochastic processes in community assembly. Many experimental studies and meta-analyses have been conducted, and the results have revealed that disturbance can lead the community structure to become more deterministic (Chase 2007) or to become more stochastic (Vellend et al. 2007),or have a neutral effect on the structure (Chase et al. 2011); for example, Chase (2007) suggested that harsh “ecological filters” reduced the importance of stochastic processes in structuring biotic communities and reduced the compositional heterogeneity by exclusion of intolerant taxa. On the other hand, Vellend et al. (2007) revealed that past agricultural land use resulted in weaker species–environment relationships in forest plant communities and reduced the importance of deterministic processes. These results suggest that differences in response of meta-community structure to disturbance depend on the type of disturbance (which species are excluded by disturbance filter?) and relative importance of processes structuring the meta-communities (which process determines compositional heterogeneity?). Under the natural conditions, meta-communities are structured by multiple processes operate at different scales. Species in local communities are selected by large-scale factors (e.g., climate, altitude) at first, and then, effect of fine-scale factors (e.g., soil condition) becomes relevant (de Bello et al. 2013a). According to earlier studies, community assembly pattern is assumed to shift from abiotically driven convergence to biotically driven divergence with decreasing spatial scales (de Bello et al. 2013b). Therefore, compositional heterogeneity along each ecological gradient may also show scale-dependent response after the change in disturbance regime. However, evidence based on observational field studies mainly focusing on relatively homogeneous meta-community with slight influence of compositional heterogeneity (Lepori and Malmqvist 2009) or overlooking hierarchy among multiple processes structuring compositional heterogeneity (McKinney 2006; Donohue et al. 2009; Matthiessen et al. 2010). An improved understanding of differences in the relationship between disturbance and the responses of the assembly pattern of a heterogeneous meta-community along multiple environmental gradients could provide new insights into how ecological processes interact across scales and influence patterns of diversity.

Over the last century, anthropogenic drivers have caused drastic changes in the population dynamics and habitat usage of large herbivores. Some of these herbivores have increased in range and population at national scales (Newson et al. 2012) and have become major drivers responsible for drastic changes in plant communities (e.g., Côté et al. 2004). Large herbivores cause disturbance by selective foraging and trampling of vegetation and thereby modify the pattern of relative abundance of plant species within local communities (e.g., Ohashi et al. 2007). In addition, large herbivores may act as agents of long-distance dispersal via epi-and endozoochory, which promote the interchange of propagules among local communities (Auffret et al. 2012). Moreover, several studies have reported that an increase in the disturbance caused by large herbivores has driven biotic homogenization by causing a loss of palatable habitat specialists and the expansion of browse-tolerant generalists (McKinney and Lockwood 1999; Rooney et al. 2004; Weigmann and Waller 2006; Rogers et al. 2008; Rooney 2009). The results of these studies suggest that intensification of the disturbance by large herbivores may alter the assembly pattern of meta-communities and influence the pattern of species diversity at a regional scale.

**Figure 1:**
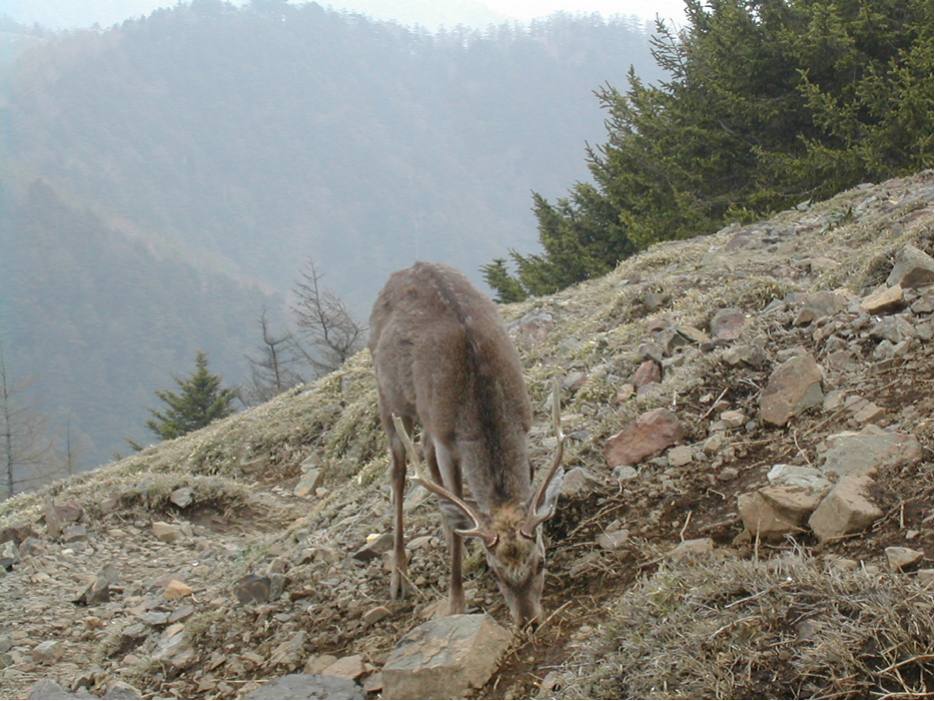
Photo of Sika deer (*Cervus nippon*Temminck) in Chichibu-Tama-Kai National Park.

Overbrowsing by sika deer (*Cervus nippon* Temminck; Fig. 1) has been reported to cause serious impacts throughout Japan, including in more than half of the country's 83 national parks (Tokida 2006). Damage control has been ineffective because of unexpectedly rapid and large-scale population increases and range expansion, while the hunter population is increasingly aging (Takatsuki 2009). Deer browsing is now becoming a major threat to biodiversity in Japan. As the area with heavy browsing pressure is expanding at a national scale (Planning Committee, The Society of Vegetation Science 2011), it is important to clarify how the current overabundance of sika deer will alter the assembly pattern of meta-communities and affect species diversity across multiple spatial scales. Thus, our aim in this study was to examine whether an intensification of disturbance by sika deer would affect the community assembly pattern in heterogeneous meta-communities. If our analysis revealed a significant influence, our second aim was to determine whether the direction of change differed among the ecological gradients that create variation in the species composition of the local community.

## Materials and Methods

### Data collection

To quantify changes in the community assembly pattern, we resurveyed the species composition of vascular plants in plots distributed across ca. 110 km^2^ of the eastern part of Chichibu-Tama-Kai National Park (35°41.1′N to 36°2.1′N, 138°30.0′E to 139°19.2′E) in central Japan. The mean annual temperature was 11.9°C, with a minimum mean monthly temperature of 1.3°C in January and a maximum mean monthly temperature of 23.2°C in August, and mean annual precipitation was 1623.5 mm, with a humid summer and a dry winter (Ogouchi weather station, 35°47.5′N, 139°3.5′E, 530 m a.s.l., 1981 to 2010; http://www.jma.go.jp/jma/index.html, accessed May 2013). This region is characterized by complex topography, with steep slopes of more than 30° on Paleozoic and Mesozoic sedimentary rock. In this region, sika deer populations increased drastically in the late 1990s. The population density was 1.9 ± 1.2 (mean ± SD) deer/km^2^ in 1987 (Educational Board of Gunma Prefecture et al. 1988), but had increased to 11.6 ± 12.5 deer/km^2^ by 2002 (Japan Wildlife Research Center 2003).

The survey plots were initially surveyed from 1979 to 1985 (Okutomi et al. 1987; Oono 1987), with the main aim being to provide a classification of the plant communities in this region. Within the >200 original plots, we were able to relocate 92 plots with elevations ranging from 750 to 2015 m a.s.l. for a second survey conducted from 1999 to 2006. To locate the original survey plots, we used their coordinates drawn from maps at a scale of 1:25,000, the slope and slope aspect, the species composition of the tree-crown layer, and sketches of each plot that showed the tree trunks and other structures. The plots included two types of grassland communities and five types of forest communities. The area of the survey plots ranged from 3 to 16 m^2^ in the grassland communities and from 100 to 400 m^2^ in the forest communities. In a previous study in this region, significant changes in species composition were observed between the 1979–1985 and 1999–2006 surveys, and the changes were attributed to increases in the population density of sika deer (Ohashi et al. 2007).

### Environmental and spatial variables

We analyzed three major environmental variables which considered as potentially affect species composition of plant community in the survey area (Okutomi et al. 1987). Altitude (*ALT*) was considered as a variable acting at the largest spatial scale in the study area. We determined *ALT* from a digital elevation model (*DEM*) at 10-m resolution (Geospatial Information Authority of Japan, http://www.gsi.go.jp/kiban/index.html, accessed June 2012). Topographic position index (*TPI*, Gallant and Wilson 2000) was also determined from same digital elevation model. *TPI* was considered as a variable acting at intermediate spatial scale. *TPI* measures the relative topographic position of the central point as the difference between the elevation at this point and the mean elevation within a predetermined radius. In this study, we predetermined the radius as 50 m. *TPI* was calculated using the focal operators of ArcGIS 10.0 (ESRI Inc., Redlands, CA). We also treated the difference in habitat (grassland vs. forest) as an environmental variable. This variable was considered as a variable acting at the most fine spatial scale.

Spatial variables were generated using the principal coordinates of neighbor matrices (PCNM) method (Borcard and Legendre 2002; Dray et al. 2006). This method enables to detect and quantify the spatial structure at multiple scales. The PCNM variables are a suite of orthogonal variables that are ordered based on the spatial scales they represent. These variables represent spatial information as an object-by-variable matrix, a form compatible with applications of multiple regression or canonical ordination (RDA or CCA). The PCNM variables are obtained by eigenvalue decomposition of a truncated matrix of geographic distances among the sampling sites. At first, we generated a Euclidean distance matrix for the survey plots. This matrix was then truncated using a threshold value (the length of the longest edge of the minimum spanning tree that connected the survey plots), and all distances greater than this threshold were replaced by an arbitrary large distance. Therefore, any value larger than the threshold would serve equally by a large constant value. The value arbitrarily used in calculation was four times the value of the threshold. Finally, we calculated the principal coordinates of this matrix. Because the eigenvalues of the matrix are equal to Moran's *I* coefficients of spatial autocorrelation, they can be either positive or negative. We used variables with a positive value of Moran's *I* value as spatial explanatory variables (Dray et al. 2006).

### Statistical analysis

#### Change in nonrandomness of community assembly pattern

To test the significance of changes in the community assembly pattern between the two survey periods, we used a null model approach proposed by Chase et al. (2011). This approach allows to calculate metric of compositional turnover independent from the effect of local (*α*-) and regional (*γ*-) diversity, which are majority of dissimilarity metrics strongly influenced by. We calculated the Raup–Crick metric as an indicator of the dissimilarity in species composition between pairs of communities (Raup and Crick 1979). Raup–Crick metric can provide information on the degree to which pairs of communities are more different (or more similar) than null expectation from difference in *α*-diversity among locality, based on 9999 randomizations (Chase et al. 2011). This metric was calculated by following steps (Chase et al. 2011):

1. Calculate the number of species observed in each site (*α*_*1*_, *α*_*2*_) and number of species that the two sites share in common (*SS*_*obs*_).
2. Calculate the total number of species in the all sites in the data set of interest (*species pool*) and the proportion of sites each species occupies (*occupancy*).
3. To calculate the distribution of *SS*_*exp*_ values, randomly draw *α*_*1*_ and *α*_*2*_ species at random from the *species pool*. The probability of a species being drawn is proportional to its *occupancy*.
4. To compare the *SS*_*obs*_ with the distribution of *SS*_*exp*_, sum the number of random draws which *SS*_*exp*_ > *SS*_*obs*_ and one-half of the random draws in which *SS*_*obs*_ = *SS*_*exp*_ and divide the sum by the total number of random draws. Repeat this procedure 9999 times.
5. To standardize the metric to range from −1 to 1, subtract 0.5 from the value from step 4 and multiply 2.

A value will be close to 0 when community assembly pattern is random and represents no difference in the observed dissimilarity from the null expectation; a value will be close to 1 when an observed dissimilarity higher than the value expected by chance, and a value will be close to −1 when an observed dissimilarity less than the value expected by chance. Value close to −1 or 1 indicates contribution of nonrandom process (e.g., deterministic environmental filters) creating highly dissimilar (or highly similar) local communities.

We compared the frequency distributions of the Raup–Crick metric from the first and second surveys. We used Wilcoxon's signed rank test to test significance of change in absolute value of a metric and determined whether the assembly pattern of the communities became nonrandom (i.e., a Raup–Crick metric closer to 1 or −1) or more random (a metric closer to 0). We also used Wilcoxon's signed rank test to test significance of change in raw value of Raup–Crick metric and determined whether the communities became more homogenized (i.e., a Raup–Crick metric decreased) or more diversified (a metric increased).

#### Change in contribution of environmental and spatial factors

To quantify the contribution of the environmental and spatial factors to the species composition within each survey period, we performed variation partitioning based on canonical correspondence analysis (CCA; Borcard et al. 1992). We separately performed forward selection of the variables to ensure that only significant variables were included in the final models. We then performed partial CCA to obtain the unique contributions of each environmental and spatial factor alone, and the combined contribution of the environmental and spatial factors, after performing the correction proposed by Peres-Neto et al. (2006).

#### Change in turnover rate along environmental and spatial gradients

We quantified turnover rates of community composition along environmental and spatial gradients by calculating the slopes of the linear regressions of Jaccard's similarity index (the number of shared species between a pair of plots divided by the total number of species in a pair of plots, Magurran and McGill 2011) against environmental and spatial distance (Nekola and White 1999). To test the significance of changes in turnover rates of community composition along an ecological gradient between the two survey periods, we fitted generalized linear mixed models (GLMMs) with a Poisson error distribution and a log link function. We specified the number of shared species between pairs of plots as the response variable and the total number of species in a pair of plots as the offset, and we included two survey periods (1979–1985 vs. 1999–2006), the difference in habitat (within-habitat or between-habitat), the distance along three continuous ecological gradients (altitude, topography, spatial), and the interaction between the survey period and the other factors in the linear predictor during model fitting. We specified the identity of each pair of plots as a random effect. We followed multiple mantel test procedure based on 9999 permutation to examine whether each coefficient significantly different from zero (Manly 2007).

#### Change in niche breadth and niche overlap

Finally, we compared the difference in niche breadth and niche overlap of along the three environmental gradients between the two survey periods. We calculated niche breadth and niche overlap values for species occurred at more than 5% of total number of surveyed plot (>4 plots).

For binary variable (habitat: grassland vs, forest), we defined the habitat specialist by binomial test (*P* < 0.05). At first, species with significantly higher occurrence in specific habitat (grassland or forest) than expected from sample's proportion (grassland: 37 vs. forest: 55 plot) was defined as specialist. Then, we tested significance of difference of proportion of specialist between 1979–1985 and 1999–2006 by fisher's exact test.

We calculated niche overlap (NO) of habitat variable by the method proposed by Geange et al. (2011). Niche overlap between species *i* and *j* was calculated as:


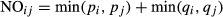


where species *i* has probability *p*_*i*_ for “occurrence in grassland” and *q*_*i*_ = 1 – *p*_*i*_ for “occurrence in forest.” Significance of difference in mean and variance of niche overlap between two survey periods was tested by randomization test.

For continuous variables (topographic position and altitude), we defined the niche breadth as the range of 95% occurrence of each species (difference between 2.5% percentile and 97.5% percentile) and tested the significance of difference between 1979–1985 and 1999–2006 by Mann–Whitney *U*-test.

We calculated niche overlap along topographic position and altitude gradient based on nonparametric kernel density function (NO_*K*_) (Geange et al. 2011). Niche overlap between species *i* and *j* was calculated as:


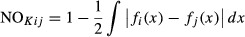


where *f*_*i*_ and *f*_*j*_ are kernel density functions for species *i* and *j*, respectively. Significance of difference in mean and variance of niche overlap between two survey periods was tested by randomization test based on 9999 permutation.

All statistical analyses were performed using version 2.15.2 of the R statistical software (R Development Core Team 2012).

## Results

### Change in nonrandomness of community assembly pattern

**Figure 2:**
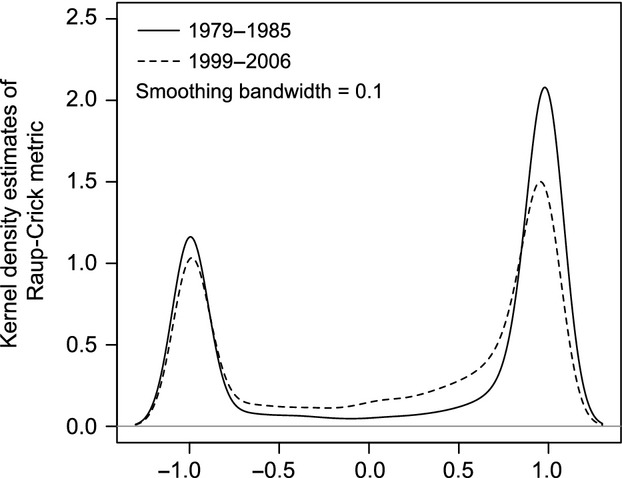
Kernel density estimates of the Raup–Crick metric between all possible pairs of survey plots (*n* = 4186) for the periods from 1979 to 1985 (solid line) and from 1999 to 2006 (dashed line).

From 1979 to 1985, the frequency distribution of the Raup–Crick metric between pairs of communities showed a bimodal distribution, with modes at −1 and 1. However, the frequency distribution from 1999 to 2006 showed a more gradual change in the distribution between these extremes, with a decreased frequency at both modes (Fig. 2). Both raw and absolute values of the Raup–Crick metric significantly decreased (raw value, *n* = 4186, *V* = 3083300, *P* < 0.001; absolute value, *n* = 4186, *V* = 3602893, *P* < 0.001).

### Change in contribution of environmental and spatial factors

**Figure 3:**
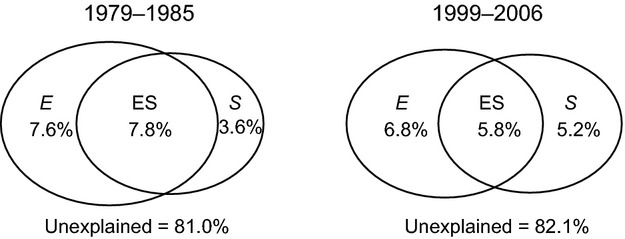
Partitioning of the variation in species composition of the vascular plant meta-community in Chichibu-Tama-Kai National Park from 1979 to 1985 and from 1999 to 2006. Values shown in the diagrams are the percentages of the variation explained exclusively by environmental conditions (E), spatial structure (S), and by the interactions between these components (ES).

The partitioning of variation based on the 1979 to 1985 data, obtained prior to intensification of the deer impact, showed that the unique contributions of the environment and space were 7.6% and 3.6%, 7.8% for the combined contribution of the environmental and spatial factors, and 81.0% for the unexplained variation. However, based on the data from 1999 to 2006, after the intensification of deer impact occurred, the unique contributions of the environmental and spatial factors were 6.8% and 5.2%, 5.8% for the contribution of the spatially structured environment, and 82.1% for the unexplained variation (Fig. 3, Appendix S1). Due to the weakened contribution of the environmental factor and strengthened contribution of the spatial factor, the ratio of the importance of spatial to environmental variation changed from 0.47 to 0.76.

### Change in turnover rate along environmental and spatial gradients

The generalized linear mixed models revealed that the similarity between pairs of local communities decreased along all ecological gradients (Table 1). The difference between the two survey periods was not significant as a single factor. However, the response of the turnover rate of community composition differed among ecological gradients. The slopes of the linear regressions of Jaccard's similarity index decreased along the habitat and topographic gradients, whereas no significant change was observed in the altitude and spatial gradients (Table 1).

**Table 1:** Results of the generalized linear mixed model analysis of difference in compositional turnover rate over environmental distance and spatial distance between two survey periods. *P*-values are from randomization test based on 9999 permutations.

Explanatory variables	Regression coefficients	SD	*P*-value
(Intercept)	−0.9458	0.0483	***
Altitude	−0.0020	0.0001	***
Habitat (between-habitat)	−1.7575	0.0319	***
Topographic index	−0.0691	0.0042	***
Spatial	−0.0141	0.0025	**
Survey period: 1999–2006	−0.1472	0.0259	NS
Altitude × 1999–2006	−0.0001	0.0001	NS
Habitat × 1999–2006	0.2007	0.0252	***
Topography × 1999–2006	0.0268	0.0029	***
Spatial × 1999–2006	−0.0045	0.0015	NS

NS, not significant

** , significance at *P* < 0.01;

*** , significance at *P* < 0.001.

### Change in niche breadth and niche overlap

**Figure 4:**
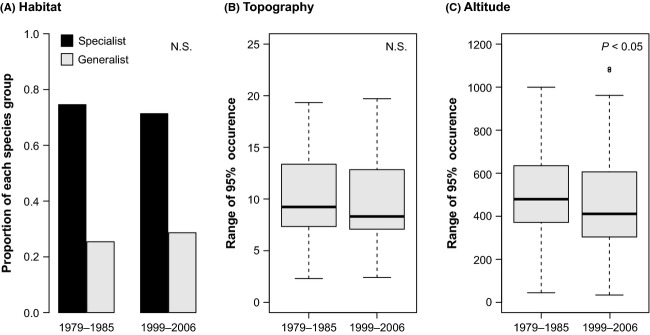
(A) Proportions of habitat specialists and generalists from 1979 to 1985 and from 1999 to 2006. (B) Mean niche breadth along topographic gradient from 1979 to 1985 and from 1999 to 2006. (C) Mean niche breadth along altitudinal gradient from 1979 to 1985 and from 1999 to 2006.

Between the two survey periods, proportion of specialist along the habitat gradient slightly decreased from 74.6% to 71.3%, while proportion of generalist increased from 25.4% to 28.7%, although its difference was not statistically significant (Fig. 4A, Fisher's exact test, *P* > 0.10). Niche breadth along topographic gradient also did not show significant change between two survey periods (Fig. 4B, Mann–Whitney *U*-test, *P* > 0.10), while niche breadth along altitudinal gradient significantly decreased from 1979–1985 to 1999–2006 (Fig. 4C, Mann–Whitney *U*-test, *P* < 0.05).

Mean and variance of niche overlap along habitat gradient and altitudinal gradient significantly decreased from 1979–1985 to 1999–2006 (Table 2, randomization test, *P* < 0.001). On the other hand, mean niche overlap along topographic gradients significantly increased (Table 2, randomization test, *P* < 0.001), and variance of niche overlap did not show significant difference between two survey periods (Table 2, randomization test, *P* > 0.10). Coefficient of heterogeneity decreased in habitat and altitudinal gradient, while increased in topographic gradient (Table 2).

**Table 2:** Mean and variance of niche overlap along three environmental gradients, and the coefficient of heterogeneity (*η*) in 1979–1985 and 1999–2006. *P*-values are from randomization test based on 9999 permutations.

	Mean		*P*	Variance		*P*	*η*	
	1979–1985	1999–2006		1979–1985	1999–2006		1979–1985	1999–2006
Habitat	0.578	0.543	***	0.180	0.178	**	0.739	0.716
Topography	0.584	0.589	**	0.044	0.044	NS	0.181	0.182
Altitude	0.402	0.387	***	0.068	0.066	***	0.285	0.279

NS, not significant

** , significance at *P* < 0.01;

*** , *P* < 0.001.

## Discussion

### Change in nonrandomness of community assembly pattern

Our study revealed that the intensification of disturbance caused by an overabundance of large herbivores altered the assembly pattern of the meta-community in our Japanese study region. In the 1979 to 1985 period, the meta-community structure in the study region was nonrandom, as indicated by the bimodal distribution of the Raup–Crick metric (Fig. 2). However, in the 1999 to 2006 period, the distribution of the Raup–Crick metric shifted toward a more uniform distribution, with decreased peak frequencies at −1 and 1 and absolute value, which indicates a more random assembly pattern (Fig. 2). In addition, raw value of the Raup–Crick metric also decreased significantly, which indicates homogenization of the community composition. The intensification of disturbance caused by large herbivores sometimes leads to a loss of habitat specialists and expansion of the distribution of habitat generalists, resulting in a loss of the regional biotic distinctiveness and causing a form of biodiversity loss known as biotic homogenization (Rooney et al. 2004; Weigmann and Waller 2006; Rooney 2009). Although previous research had only demonstrated such biotic homogenization in a relatively homogeneous forest community, our study demonstrated that even in a heterogeneous meta-community that included both grassland and forest habitats, disturbance by large herbivores can alter the meta-community structure and cause biotic homogenization.

### Change in contribution of environmental and spatial factors

In two survey periods, both environmental and spatial factors were selected to explain the compositional variation in CCA by forward selection procedure, which suggests that species sorting and dispersal processes were working together (Cottenie 2005). However, the relative importance of the environmental and spatial factors changed between 1979 to 1985 and 1999 to 2006. The relative importance of environmental factors decreased and the relative importance of spatial factors increased (Fig. 3). This suggests that the intensification of deer disturbance had weakened the effect of environmental processes, while strengthening the effects of dispersal processes. The compositional variation among local communities is generally produced by a simple decrease in the environmental similarity under conditions of sufficient dispersal, and the species composition can then be predicted from the environment. When dispersal limitation occurs and species do not disperse to all suitable sites, current environmental conditions cannot fully explain the species distribution or the local community composition (Nekola and White 1999). Because deer browsing affects the reproductive ability of palatable species and decreases their propagule pressure (i.e., their ability to disperse to other potentially suitable sites; Anderson 1994), the intensification of deer disturbance may cause insufficient dispersal of vulnerable species and decrease the relative importance of the species–environment relationship. Moreover, disturbance by large herbivores can act as an ecological filter that selects disturbance-tolerant species, mediates the competitive ability of dominant species, creates small patches, and increases the opportunity for establishment of subdominant species (Augustine and McNaughton 1998; Suzuki et al. 2013). This may lead to propagule establishment by random chance, which would increase the stochastic processes related to dispersal.

### Change in turnover rate, niche breadth, and niche overlap along environmental gradients

In our study, the turnover rate of community composition along the ecological gradients responded differently to the intensification of deer disturbance. The compositional turnover along the forest–grassland and topographic gradients decreased after intensification of deer disturbance, whereas the compositional turnover along the altitude and spatial gradients did not change significantly (Table 1). The species composition of local communities is generally structured by a multidimensional filter that operates at different scales. An abiotic regime that operates at a large scale (e.g., climate) selects for species with a specific physiology or life history and leads to a convergence of traits in the surviving species, whereas competition for resources at a local scale (e.g., light, nutrients, water) can cause trait divergence (Grime 2006). Our study revealed that the intensification of deer disturbance altered the compositional variation created by environmental factors operating at a local scale, but did not alter the compositional variation created by factors operating at larger scales.

Generally, the wider the average niche breadth and overlap, the lower the rate of species turnover will occur over a fixed amount of environmental distance, and vice versa (Nekola and White 1999). The result of our study showed variant response of niche breadth and niche overlap among three environmental gradients. Difference in the response of niche breadth and niche overlap might be the one explanation for the heterogeneous response of the turnover rate along environmental gradients to disturbance (Table 1).

Along the habitat gradient, mean and variance of niche overlap significantly decreased (Table 2). Although it was not statistically significant, proportion of specialist showed tendency to decrease (Fig. 4A), as consistent with a previous study (Rooney et al. 2004). This result indicates that decrease in relative importance of specialist resulted in decrease in high niche overlap within specific habitat (grassland or forest), and species tend to distribute more evenly along habitat gradient. At fine scales, biotic interaction plays an important role on community assembly process. The herbivory almost always places a plant at a competitive disadvantage (Crawley 1997) because of the carbon and resource costs of losing biomass. Generally, the specialist is a better competitor for its narrower range of resources, while the generalist is a relatively worse competitor but can coexist by consuming other types of resources (Chase and Leibold 2002). When resources are locally heterogeneously distributed, niche partitioning allows coexistence of different specialist species. Increase in deer disturbance might have reduced competitive ability of specialist species at fine scale and allowed establishment of generalist species, which lead to decrease in mean and variance of niche overlap, and turnover rate along habitat gradient.

Conversely, mean niche breadth, mean, and variance of niche overlap have significantly decreased along the altitudinal gradient in 1999–2006. In other words, species in meta-communities narrowed their realized niche along altitudinal gradient after increase in deer disturbance and also resulted in decrease in niche overlap, and no significant change has observed in turnover rate along altitudinal gradient. Because plant defense and tolerant level have dependency on environmental condition such as resource availability or successional stage (Häsler et al. 2008; Hakes and Cronin 2011), response after herbivore disturbance may depend on its habitat condition. One explanation of our result is that disturbance by herbivore might have caused reduction in competitive ability and survival rate at less suitable habitat, and many species may have survived only at their most physiologically suitable habitat, in terms of altitude; that is, even if the intensification of disturbance have caused, niche differentiation along altitudinal gradient may counterbalance the homogenization and slow down the loss of regional species pool. We could not find same phenomena along the topographic gradient. Mean niche overlap significantly increased (Table 2), while variance of niche overlaps and mean niche breadth did not show significant change, and resulted in decrease in turnover rate (Table 1). Therefore, whether homogenization occurs or counterbalanced by niche differentiation after the disturbance might be scale dependent. Although scale-dependent heterogeneous response of turnover rate, niche breadth, and niche overlap was not pointed out from previous studies, it is important to understand and predict meta-community dynamics after change in disturbance regime.

## Conclusions

This study provided evidence that the intensification of disturbance caused by the overabundance of a large herbivore weakened the nonrandom assembly pattern and lead to a more random pattern related to spatial processes. These changes caused a loss of biotic distinctiveness among the local communities, leading to a loss of floristic diversity at a regional scale. However, response of turnover rate, mean niche breadth, mean, and variance of niche overlap differed among environmental gradients, depending on its scale. Our results emphasize the importance of conserving habitat specialists that represent aspects of the local environment such as habitat and topography at various altitudinal ranges, when the goal is to maintain biodiversity at a regional scale despite an overabundance of large herbivores.

## Supporting Information

Appendix S1 Canonical correspondence analysis for the partitioning of the variation in plant community composition into fractions explained by the environmental and spatial variables for the 1979–1985 and 1999–2006 survey periods.
