# Supplementary material for: Disturbance by large herbivores alters the relative importance of the ecological processes that influence the assembly pattern in heterogeneous meta-communities
Source: Ecol Evol. 2014 Feb 17;4(6):766–75. doi: 10.1002/ece3.987 (PMC3967902; doi:10.1002/ece3.987)
Supplement: Appendix S1 — Canonical correspondence analysis for the partitioning of the variation in plant community composition into fractions explained by the environmental and spatial variables for the 1979–1985 and 1999–2006 survey periods. [file ece30004-0766-sd1.docx]

Appendix S1. Canonical correspondence analysis for the partitioning of the variation in plant community composition into fractions explained by the environmental and spatial variables for the 1979–1985 and 1999–2006 survey periods. Adj., adjusted percentage of unique and combined contribution of the environmental and spatial factors.

|  | 1979–1985 | | |  | 1999–2006 | | |  |
| --- | --- | --- | --- | --- | --- | --- | --- | --- |
|  | df | Adj. % | *P* |  | df | Adj. % | *P* | |
| *Unique contribution of environmental factors* |  |  |  |  |  |  |  | |
| Habitat [H] | 1 | 3.8 | ** |  | 1 | 3.2 | ** | |
| Altitude [A] | 1 | 2.6 | ** |  | 1 | 2.1 | ** | |
| Topographic index [T] | 1 | 0.7 | ** |  | 1 | 0.6 | ** | |
| *Combined contribution of the environmental factors* | |  |  |  |  |  |  | |
| [H∩A] |  | -0.2 |  |  |  | -0.1 |  | |
| [H∩T] |  | 0.7 |  |  |  | 0.5 |  | |
| [A∩T] |  | 0.0 |  |  |  | 0.0 |  | |
| [H∩A∩T] |  | 0.1 |  |  |  | 0.5 |  | |
| *Unique contribution of the spatial factors* |  |  |  |  |  |  |  | |
| Spatial factors [S] | 11 | 3.6 | ** |  | 11 | 5.2 | ** | |
| *Combined contribution of the environmental and spatial factors* | | | |  |  |  |  | |
| [H∩S] |  | 2.1 |  |  |  | 1.7 |  | |
| [A∩S] |  | 2.8 |  |  |  | 2.3 |  | |
| [T∩S] |  | 0.8 |  |  |  | 0.6 |  | |
| [H∩A∩S] |  | 0.9 |  |  |  | 0.6 |  | |
| [H∩T∩S] |  | 0.5 |  |  |  | 0.5 |  | |
| [A∩T∩S] |  | 0.1 |  |  |  | 0.1 |  | |
| [H∩A∩T∩S] |  | 0.6 |  |  |  | 0.0 |  | |
| 1–[H+A+T+S] |  | 81.0 |  |  |  | 82.1 |  | |
|  |  |  |  |  |  |  |  | |
| Total contribution of environmental factors (E) |  | 7.6 |  |  |  | 6.8 |  | |
| Ratio of spatial to environmental variation (S:E) |  | 0.48 |  |  |  | 0.76 |  | |
